# Three Weeks at a Seaside Convalescent Home

**Published:** 1915-02-27

**Authors:** 


### FROM THE PATIENT'S POINT OF VIEW.

(Contributions to this Section are Invited.)

### Three Weeks at a Seaside Convalescent Home.

Winter may not seem a very desirable time for a stay at a convalescent home on the coast after prolonged medical or surgical treatment, but so attractive did I find a fortnight's sojourn during January at one institution that I cannot refrain from committing to paper my impressions.

### First Impressions.

Having obtained the necessary medical certificate and order for admission, I soon found myself at the seaside railway station where a carriage with only one occupant—a country patient—was awaiting us. No sooner had we arrived than we were taken into the office of the "sister" of the men's ward, and after several questions had been put and answered we were shown by the attendant into the day room—a lofty and cosy apartment with couches and easy chairs, where, after the rules had been read to us, we were left to rest until the bell rang for tea at 4.30 o'clock. After the meal we were weighed in the surgery, and at 9 o'clock retired to rest.

A fine red-brick building standing in its own grounds, and with a certain ecclesiastical aspect, it is very near to the seafront. The town I found "alive" with innumerable recruits for Kitchener's new Army; but, alas! their marches were without that band for which Mr. Rudyard Kipling and others have recently so earnestly pleaded. Many British and Belgian wounded soldiers were to be seen recuperating, especially along the seafront.

A day or two later we (myself and carriage companion) were seen by the doctor of the institution. We soon became accustomed to the new mode of life. The general harmony which existed in the place—due to the delightful bearing and cheery manner of the sisters—was apparent from the hour of admission, and was a very pleasing feature in the life of the place. The patients certainly did seem to appreciate the pains which were bestowed to make them thoroughly comfortable, and not a single word of adverse criticism, or the slightest murmuring or complaint, was uttered by any of them during my stay. Indeed, the Apostolic injunction, "Bear ye one another's burdens," was admirably displayed by the inmates—mainly of the artisan class—especially as regarded behaviour towards the cripples.

As to the day's routine. At 7 o'clock the patients rose, washed, and assembled in the day room, where a few minutes before the breakfast hour—8 o'clock—the chaplain entered and read prayers. After breakfast, a substantial one, which served as a sort of armour for the very weak patients who, in their morning strolls, would have to contend with the strong (if not to them devouring) breezes, the "sister" served out medicine in the surgery to those patients for whom it had been prescribed by the doctor.

### No Indoor Duties.

Until dinner—12.30 o'clock—the men were free to dispose of their time. No indoor domestic duties were set them, and this exemption, even from a little light toil, was greatly appreciated as they seemed so much to value every hour of their time at this "Empress of Watering Places." To enter a public-house was, of course, strictly forbidden, and there were no offenders in respect to this rule. The seafront was the favourite resort of the patients, and here when tired the shelters provided at intervals proved extremely welcome. Not all were strong enough to climb to the summit of ——, some 550 feet above sea-level, which reminded one, although, of course, in a diminutive way, of Table Mountain, as seen when a ship lies becalmed off the Cape of Good Hope.

### The Sergeant's Advice.

Dinner was served at 12.30 o'clock to the patients, numbering under twenty, an unusually small number for the time of year. The food, necessarily always an important factor at convalescent institutions, was excellent in quality and abundant, and, with one or two exceptions, through the nature of the patient's illness, all were able to give their best attention to it. Certainly the exhilarating air was entirely responsible for the inmates finding that they had not left their appetites at home. As a matter of fact, the good "sergeant," who exercised a kind of paternal care over the patients, exhorted them very carefully, after reading the rules on their admission, "to eat well, but to waste nothing." Presumably he had in mind the weighing scales which awaited every man the day before exit, and was anxious that they should not then bring discredit upon him by being "found wanting" on his scales.

After dinner the morning procedure would, in the main, be repeated by those who were able to take outdoor exercise. Tea would be served soon after 4.30 o'clock, and no patients would be allowed out afterwards. Of course, this rule does not apply to other seasons of the year.

### The "Smoke-hole."

After tea the patients would retire to the day room or bagatelle room adjoining, or indulge in smoking in the garden "retreat." At 6.30 o'clock there would be Evensong in the beautiful chapel connected with, and forming part of, the building. On Sundays the patients were required to attend both morning and evening. At 7.15 o'clock the supper-bell would ring. After the meal the patients would again amuse themselves in the day room, or go across to the octagonal-shaped *fumoir* at the further end of the garden, known among the inmates as the "smoke-hole." The large cigar-shaped door-handle and the ornamental ironwork on its panels resembling churchwarden pipes suggest very strikingly the character of the place. The "hole" is, it is needless to say, the only place where the pipe or cigarette is tolerated.

As 9 o'clock draws near the smokers leave their retreat, rejoin their more sedate companions around the comfortable fire in the day room, and await the last ring of the bell. The lift, which is in the centre of the handsome entrance hall and close to the staircase, is used mainly by the cripples to go to the dormitories. Over the entrance to the wards on each landing is, in scroll-like form, an appropriate quotation from the sacred Scripture. The sergeant—an admirable fellow with some naval experience, by the way—sees that the lofty curtained windows are perfectly closed, so as not to allow of any possible draughts, ignites the floating nightlight which affords just a glimmer in the capacious wards during the hours of darkness, switches off the electric light, and with a cheery "Good night, gentlemen all," retires.

It is scarcely possible for patients to return to their daily toil at the bench or desk without having pleasant and grateful memories of their convalescence.

